# Computational comparative analysis identifies potential stemness-related markers for mesenchymal stromal/stem cells

**DOI:** 10.3389/fcell.2023.1065050

**Published:** 2023-03-01

**Authors:** Myret Ghabriel, Ahmed El Hosseiny, Ahmed Moustafa, Asma Amleh

**Affiliations:** ^1^ Biotechnology Graduate Program, American University in Cairo, New Cairo, Egypt; ^2^ Department of Biology, American University in Cairo, New Cairo, Egypt; ^3^ Systems Genomics Laboratory, American University in Cairo, New Cairo, Egypt

**Keywords:** mesenchymal stem cells, proteasome, stemness-related markers, transcriptomics, gene interaction networks

## Abstract

Mesenchymal stromal/stem cells (MSCs) are multipotent cells that reside in multiple tissues are capable of self-renewal and differentiation into various cell types. These properties make them promising candidates for regenerative therapies. MSC identification is critical in yielding pure populations for successful therapeutic applications; however, the criteria for MSC identification proposed by the International Society for Cellular Therapy (ISCT) are inconsistent across different tissue sources. This study aimed to identify potential markers to be used together with the ISCT criteria to provide a more accurate means of MSC identification. Thus, we carried out a computational comparative analysis of the gene expression in human and mouse MSCs derived from multiple tissues to identify the differentially expressed genes that are shared between the two species. We show that six members of the proteasome degradation system are similarly expressed across MSCs derived from bone marrow, adipose tissue, amnion, and umbilical cord. Additionally, with the help of predictive models, we found that the expression profile of these genes correctly validated the identity of the MSCs across all the tissue sources tested. Moreover, using genetic interaction networks, we showed a possible link between these genes and antioxidant enzymes in the MSC antioxidant defense system, thereby pointing to their potential role in prolonging the life span of MSCs. According to our findings, members of the proteasome degradation system may serve as stemness-related markers.

## Introduction

Mesenchymal stem cells (MSCs) are multipotent adult stem cells that can be isolated from a variety of tissues such as bone marrow (BM) (Friedenstein et al., 1968), adipose tissue (AT) (Zuk et al., 2002), amnion (AM) (Alviano et al., 2007), umbilical cord (UC) (Erices et al., 2000). Due to the myriad sources of MSCs, the International Society for Cellular Therapy (ISCT) proposed minimum criteria by which these MSCs can be identified. These criteria include 1) plastic-adherence of cells *in vitro*, 2) expression of specific cell surface markers (CD105, CD90, and CD73), and lack of expression of others (CD45, CD14, CD19, CD34, CD11b, CD79alpha, and HLA-DR), and 3) ability to differentiate into osteoblasts, chondroblasts, and adipocytes *in vitro* (Dominici et al., 2006). Unfortunately, growing evidence shows that these criteria are not consistent across different tissues and species since they define only general functional and morphological characteristics (Peister et al., 2004). As a result, scientists have resorted to using additional “stemness” or “stemness-related” genes as markers to aid in the correct identification of MSCs (Zhao et al., 2017). Proper identification of MSCs is crucial to producing pure populations, thereby increasing their use in regenerative therapies. Thus, MSCs are an attractive tool for regenerative therapies, as their ease of isolation and ability to differentiate into multiple lineages make them ideal candidates for this purpose.

MSCs play a critical role in tissue maintenance, regeneration, and homeostasis *in vivo* (Minguell et al., 2001). Generally, MSCs remain quiescent, relying on glycolysis to produce energy for their metabolic needs (Sart et al., 2015); however, upon tissue injury or loss, MSCs are activated to regenerate the damaged tissue and exit quiescence in favor of a more proliferative state. This highly proliferative state must maintain the balance between replenishing downstream lineages and replenishing the stem cell pool. As they begin to proliferate, energy demands increase, and glycolysis shifts to oxidative phosphorylation; this shift is accompanied by an increase in the production of reactive oxygen species (ROS) (Liang and Ghaffari, 2014). Oxidative phosphorylation is, indeed, a much more efficient means of generating ATP than glycolysis and can produce up to fifteen times more ATP. However, this is a double-edged sword since excess ROS can impair self-renewal and proliferation of MSCs (Ko et al., 2012; Choo et al., 2014).

For this reason, MSCs have an active antioxidant defense system. It has been demonstrated that MSCs constitutively express high levels of antioxidant enzymes, such as superoxide dismutases, catalases, and glutathione peroxidases (Valle-Prieto and Conget, 2010). These enzymes repair oxidatively damaged proteins, but some become oxidatively modified or damaged irreversibly. The cell has systems that recognize and remove these irreversibly damaged proteins and consequently prevent their buildup (Jung and Grune, 2008). One of these systems is the proteasome degradation system, which plays a vital role in the degradation of oxidized and damaged proteins, preventing their accumulation and subsequent cellular dysfunction (Chondrogianni et al., 2014).

The 26S proteasome is a multicatalytic degradation complex composed of a core particle (the 20S) and one or two regulatory particles (19S). The 20S core comprises four rings, two of which are composed of seven alpha subunits, while the other two rings are composed of seven beta subunits. The 19S regulator is comprised of a base (containing six ATPase and two non-ATPase subunits) and a lid (containing up to 10 non-ATPase subunits) (Tanaka, 2009). The proteasome’s primary function in the cell is to degrade unneeded or damaged proteins by proteolysis; this can be carried out in either a ubiquitin-dependent manner through the 26S pathway or a ubiquitin-independent way through the 20S pathway. Recently, the proteasome has gained a lot of attention, and it has been shown to play an essential role in preserving the self-renewal and stemness of human MSCs. Kapetanou and others showed that senescence and loss of stemness in human MSCs are accompanied by a sharp decline in proteasome content and activity (Kapetanou et al., 2017). Furthermore, they showed that the expression of some proteasome subunits is possibly affected by pluripotency factors such as Oct4. Taken together, these observations support their hypothesis of a relationship between proteostasis and stem cell function, where proteostasis is critical in maintaining proper protein levels, leading to efficient multipotency and self-renewal maintenance.

In this study, we carried out a comparative analysis of publicly available RNA-Seq data of MSCs derived from different tissues of origin (BM, AT, UC, AM) and various species (human, mouse) to yield a list of common differentially expressed genes (DEGs). We provide evidence that members of the proteasome show similar patterns of expression across all MSC samples. Furthermore, we offer a possible relationship between the proteasome and antioxidant enzymes in protecting MSCs from oxidative stress, highlighting their importance in MSC survival. Finally, we demonstrate that six proteasomal degradation systems can be used as supplementary stemness-related markers for MSC identification through predictive models.

## Materials and methods

### RNA-seq datasets and processing

RNA-seq datasets were obtained from the Gene Expression Omnibus (GEO) (Barrett et al., 2012) and Array Express databases (Athar et al., 2018). We collected transcriptomic data for human MSCs derived from umbilical cord (h_UC_MSCs), amnion (h_AM_MSCs), bone marrow (h_BM_MSCs), and adipose tissue (h_AT_MSCs) and their tissue-specific counterparts (h_UC_TSCs, h_AM_TSCs, h_BM_TSCs, and h_AT_TSCs). Mouse transcriptomic data included bone marrow-derived MSCs (m_BM_MSCs) and adipose tissue-derived MSCs (m_AT_MSCs), along with their tissue-specific counterparts (m_BM_TSCs and m_AT_TSCs) (Supplementary Table S1). The tissue-specific counterpart cells are all cells other than the MSCs found in the MSCs tissue of origin. Those cells served as a reference for identifying differentially expressed genes that distinguished the MSCs from the other pool of cells belonging to the tissue to allow proper comparison between the different MSCs without any interfering background from the tissue from which MSCs were derived. We processed triplicates of each cell type except for h_AM_TSCs, h_AM_MSCs, and h_UC_MSCs, for which we managed to obtain quadruplicates, bringing our total number of samples to 27 human samples and 12 mouse samples (Supplementary Table S2). We used publicly available data; every triplicate was retrieved from a different experiment and a different lab. However, we made sure that the culturing conditions of the cells were similar across all the samples. In addition, all the MSCs samples were primary cultures and sequenced at passage 3. Data for all cell types were converted from the Sequence Read Archive (SRA) format into the FASTQ format using the SRA Toolkit version 2.10.8 for downstream analysis (Leinonen et al., 2010). Moreover, data were filtered; any read with a length less than 50 bp was excluded. Adapter sequences were detected and trimmed using fastp version 0.19.5 (Chen et al., 2018).

#### RNA‐seq data analysis to find DEGs

We used Kallisto version 0.46.1 for pseudo-alignment and the quantification of abundances of transcripts from the RNA-Seq data (Bray et al., 2016). Human data were pseudo-aligned to the human reference transcriptome GCA_000001405.15_GRCh38, while mouse data were pseudo-aligned to the mouse reference transcriptome GCA_000001635.9_GRCm39 provided by the Genome Reference Consortium (O'Leary et al., 2016). Pseudomapping was performed using Kallisto (Bray et al., 2016) through the identification of transcripts that a read is compatible with and assigning it a target ID. Each target ID has a corresponding accession number in the index file. Then the abundances of the transcripts are quantified and output files of abundances containing the transcript per million (TPMs) of each target ID and their corresponding accession numbers are produced. After quantification, Sleuth version 0.29.0 was used for the differential expression analysis of the transcript quantifications between mesenchymal stem cells and their tissue specific counterparts.

Sleuth loaded the Kallisto processed data, estimated the parameters for its response error measurement “full” model followed by the estimation of the parameters for its reduced model, and performed differential analysis using the likelihood ratio test. Sleuth normalizes the data by its ability to distinguish between technical and biological variance and performs shrinkage to the model only on the biological component of variance. Sleuth accounts for technical variability in the abundance estimates and models the true abundance using a general linear model, while including the technical variance as error in the response variable. Thereby, distinguishing between technical or biological sources of variance when determining differentially expressed transcripts.

Accordingly, Sleuth produced a table of significant differentially expressed genes (DEGs) with a q value less than 0.05.This step generated lists of significant DEGs for each tissue type and species. The lists included the gene symbols of significant DEGs and their corresponding TPMs (Pimentel et al., 2017). The gene symbols of each list in the human data were compared and the common DEGS retrieved with their equivalent expression. This step was repeated for the mouse data and the common DEGs retrieved. Subsequently, the list of common DEGs identified between the human MSCs samples were cross referenced and compared to the gene symbols of the common DEGs between the mouse MSCs samples to produce a list of common DEGs between the two species with their equivalent expression. Gene symbols that weren’t common between the two species were checked for homology using Homologene (http://www.ncbi.nlm.nih.gov/homologene/) and the analysis repeated. Venn diagrams of the common DEGs were constructed using Venny version 2.0 (Oliveros, 2007). Finally, the expression of the common DEGs was compared and visualized using R Studio 3.6.1 that generated heatmaps and t-distributed stochastic neighbor-embedding clustering (www.r-project.org) (Team RStudio, 2019).

#### DEGs ontology and enrichment analysis

Biological processes encompassing the DEGs were identified based on GO enrichment analysis using the GOrilla database (Eden et al., 2009). The *p*-value threshold was set at 10e-3. Afterward, we visualized the enriched GO terms using ReViGO, and a scatter plot was produced showing the log10 *p*-value and log size of each GO term (Supek et al., 2011).

#### Generation of gene interaction network

To further investigate the interactions between the DEGs, we constructed a gene interaction network by mining interaction networks from the GEO, BioGRID, IRefIndex, and I2D using the GeneMANIA Cytoscape plugin (Montojo et al., 2010). This step produced an annotated Cytoscape network of functional interactions between the DEGs.

#### Predictive model

Finally, to assess the ability of the selected DEGs’ to identify MSCs, we built a predictive model using the Waikato Environment for Knowledge Analysis (WEKA) software version 3.8.4 (Witten et al., 2016). The gene expression values were converted into the ARFF file format, where our genes of interest were used as attributes in the training dataset. The dataset used for training and testing the model consisted of the expression levels of the 22 proteasome genes across six TSCs, six MSCs mouse samples, thirteen TSCs and fourteen MSCs human samples. We used the AutoWEKAClassifier package (https://github.com/automl/autoweka) to automatically find the best classification model for our provided dataset AutoWEKAClassifier performed 486 evaluations of its available classifiers and found random forest to be the best classifier with the best error rate for this dataset. The random forest tree classifier was used to train the model with 10-fold cross-validation method. Briefly, this method randomly divided the dataset into 10 parts; it used nine for training and reserved one for testing. This procedure is repeated multiple instances each time reserving a different part for testing. After training the Random Forest model, we created the testing dataset from the training dataset by hiding the type class in order to test its performance in predicting tissue type in both human and mouse samples. WEKA was also used for attribute selection.

## Results

### RNA-seq analysis

**FIGURE 1:**
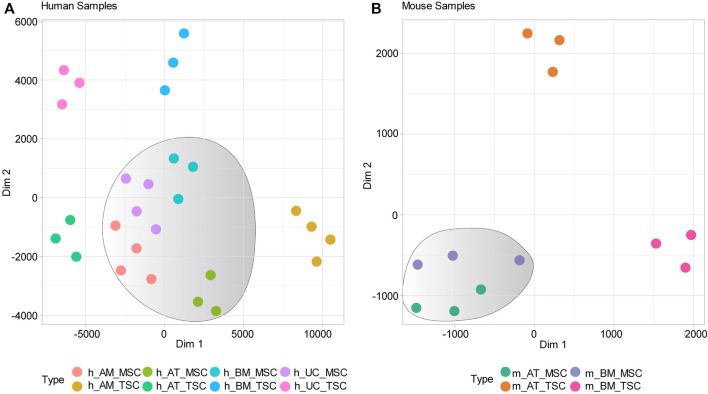
Gene expression-based clustering of MSCs samples included in the study. **(A)** and **(B)** t-SNE clustering shows distinct clusters for human and Mouse MSCs, respectively.

**FIGURE 2:**
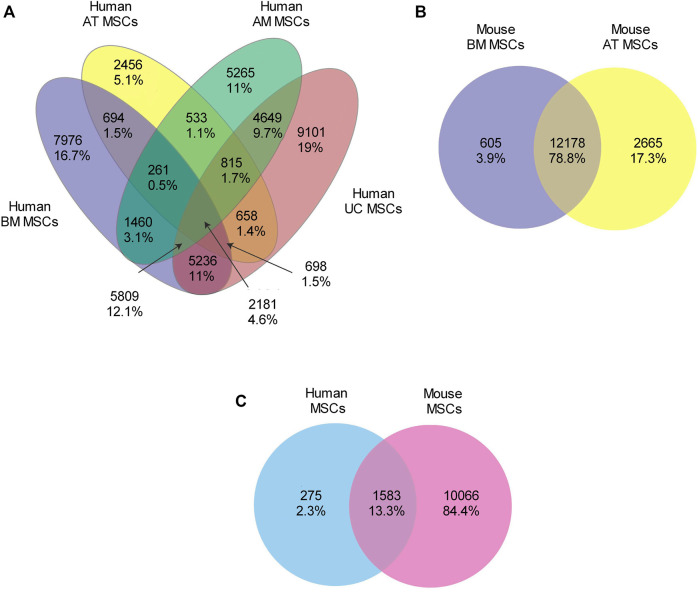
Venn diagram of the shared and unique DEGs in the transcriptomes of the MSCs derived from different tissues and species. **(A)** The shared DEGs in the transcriptomes of MSCs derived from four tissue types of human origin are 2,181 (4.6%). **(B)** The shared DEGs between the mouse BM_MSCs and the AT_MSCs are 12,178 genes (78.8%). **(C)** The common DEGs between human and mouse MSCs are 1,583 (13.3%).

We constructed clustering maps using the t-distributed stochastic neighbor embedding (t-SNE) statistical method to visualize the transcriptomic similarities and differences between the samples. The clustering maps showed that all human MSC samples clustering together in a distinct cluster apart from tissue-specific cell (TSCs) samples (Figure 1A). Likewise, the mouse MSC samples clustered together, while TSC samples clustered independently (Figure 1B). Next, we compared the gene expression of human-derived MSCs against their tissue-specific counterparts and identified 20,973, 24,365, 8,296, and 29,197 DEGs for h_AM_MSCs, h_BM_MSCs, h_AT_MSCs, and h_UC_MSCs, respectively. We constructed a Venn diagram to visualize the common DEGs shared by the MSCs derived from the four different tissue sources. The common DEGs made up 4.6% of the examined DEGs, equivalent to 2,181 common DEGs (Figure 2A) (Supplementary Table S3).

**FIGURE 3:**
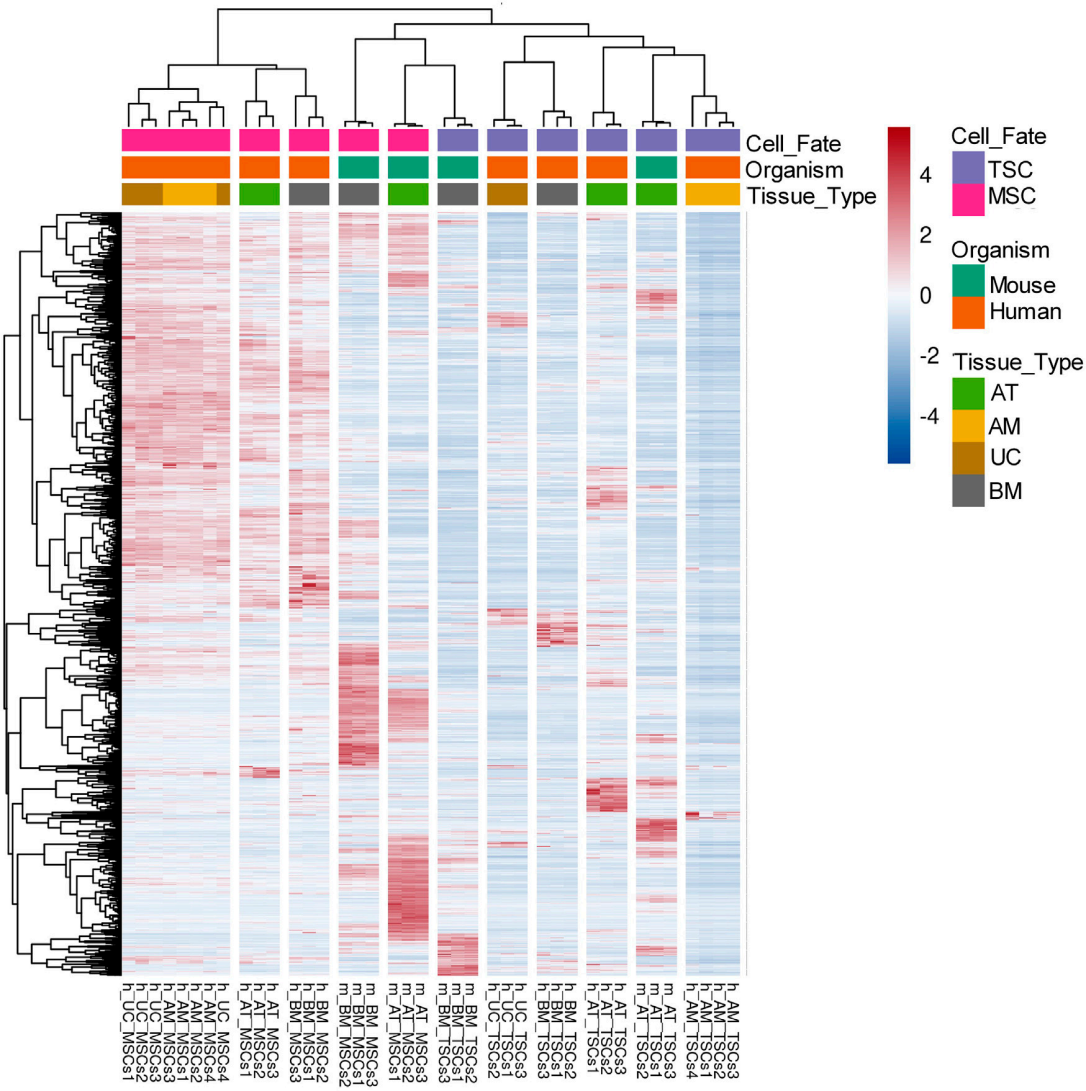
Heatmap of the DEGs across all MSCs samples. Heatmap of 1,583 MSCs DEGs between human and mouse showing three main clusters: a cluster of all human MSCs samples, a cluster of all mouse MSCs samples with m_BM_TSCs, and a cluster of all the TSCs samples. The normalized expression values are color-coded where red indicates high expression and blue indicates low expression.

Similarly, for the mouse datasets, we compared the gene expression of mouse-derived MSCs with their tissue-specific counterparts and identified 14,843 DEGs for m_AT_MSCs and 12,783 DEGs for the m_BM_MSCs. We found that the common DEGs made up 78.8% of the examined DEGs, equivalent to 12,178 common DEGs (Figure 2B; Supplementary Table S4). Finally, we compared both lists of common DEGs to determine whether the human and mouse MSCs shared any common DEGs. The Venn diagram showed that the two species had 1,583 (13.3%) DEGs in common (Figure 2C, Supplementary Table S5). Interestingly, the heatmap of the 1,583 common DEGs showed three main clusters: a cluster that included all the human MSC samples, another cluster that included all the mouse-derived MSC samples plus m_BM_TSCs, and, finally, the last cluster included the rest of the TSC samples. Each of these clusters included subclusters that grouped the triplicate of each tissue type (Figure 3).

#### DEGs ontology and enrichment analysis

In the gene ontology analysis, we produced a list of 157 enriched gene ontology (GO) terms with a threshold *p*-value of 10^–3^ (Supplementary Table S6). ReViGO generated a scatter plot of the enriched GO terms organized according to their significance (*p*-value) and uniqueness. Following visualization, we identified a unique GO term (GO:2000736) to regulate stem cell differentiation with a significant *p*-value of 8.69E-4 and a q value of 4.64E-2. Moreover, this GO term had a frequency of 0.010% and a uniqueness score of 0.70 (Supplementary Figure S1). Other GO terms were more general, less unique, and not explicitly specific to stem cell function. The GO term GO:2000736 included 23 genes that belonged to the proteasomal degradation pathway (Table1).

**TABLE 1:** List of DEGs belonging to the proteasomal degradation pathway identified in the GO enrichment analysis (GO:2000736).

HUGO ID	Systemic ID	Gene
PSMA1	α6	proteasome (prosome, macropain) subunit, alpha type, 1
PSMA5	α1	proteasome (prosome, macropain) subunit, alpha type, 5
PSMA7	α4	proteasome (prosome, macropain) subunit, alpha type, 7
PSMB1	ß6	proteasome (prosome, macropain) subunit, beta type, 1
PSMB2	ß4	proteasome (prosome, macropain) subunit, beta type, 2
PSMB3	ß3	proteasome (prosome, macropain) subunit, beta type, 3
PSMB4	ß7	proteasome (prosome, macropain) subunit, beta type, 4
PSMB5	ß5	proteasome (prosome, macropain) subunit, beta type, 5
PSMB6	ß1	proteasome (prosome, macropain) subunit, beta type, 6
PSMB7	ß2	proteasome (prosome, macropain) subunit, beta type, 7
PSMC1	Rpt2	proteasome (prosome, macropain) 26s subunit, atpase, 1
PSMC2	Rpt1	proteasome (prosome, macropain) 26s subunit, atpase, 2
PSMC4	Rpt3	proteasome (prosome, macropain) 26s subunit, atpase, 4
PSMC5	Rpt6	proteasome (prosome, macropain) 26s subunit, atpase, 5
PSMD1	Rpn2	proteasome (prosome, macropain) 26s subunit, non-atpase, 1
PSMD2	Rpn1	proteasome (prosome, macropain) 26s subunit, non-atpase, 2
PSMD3	Rpn3	proteasome (prosome, macropain) 26s subunit, non-atpase, 3
PSMD4	Rpn10	proteasome (prosome, macropain) 26s subunit, non-atpase, 4
PSMD5	Rpn4	proteasome (prosome, macropain) 26s subunit, non-atpase, 5
PSMD7	Rpn8	proteasome (prosome, macropain) 26s subunit, non-atpase, 7
PSMD8	Rpn12	proteasome (prosome, macropain) 26s subunit, non-atpase, 8
PSMD13	Rpn9	proteasome (prosome, macropain) 26s subunit, non-atpase, 13
PSMD14	Rpn11	proteasome (prosome, macropain) 26s subunit, non-atpase, 14

#### Gene expression analysis

**FIGURE 4:**
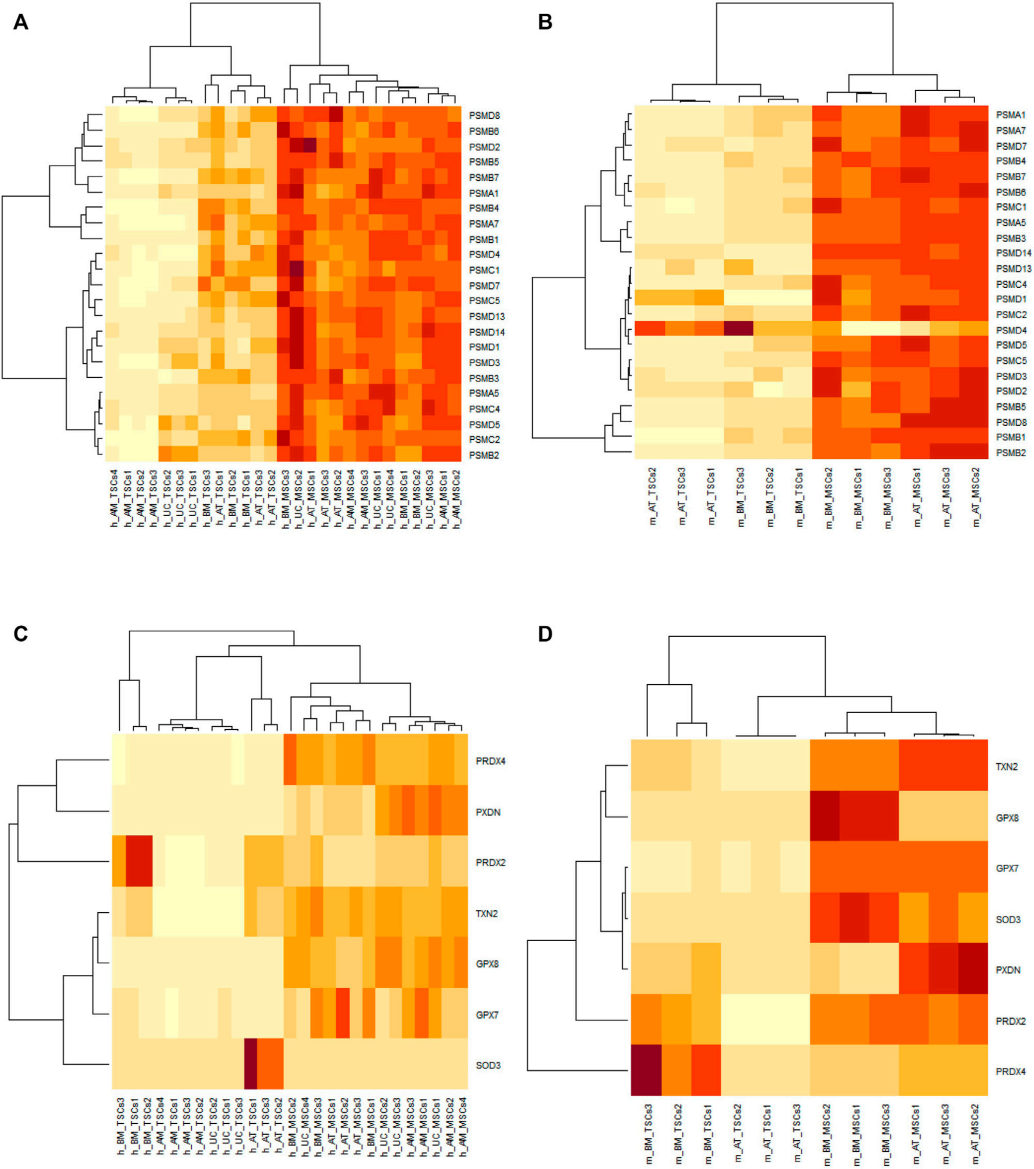
Heatmaps of DEGs between MSCs and their TSCs generated by RStudio. Orange indicates upregulation and yellow indicates downregulation. **(A)** and **(B)** heatmaps of the proteasomal genes’ expression in human and mouse samples, respectively, showing all genes to be upregulated in MSCs samples except PSMD4 in mouse MSCs. **(C)** and **(D)** heatmaps showing the expression of the antioxidant genes is upregulated in human and mouse MSCs samples, respectively, with the exception of SOD3 and PRDX2 in human AT_MSCs and BM_MSCs, and PRDX4 in mouse BM_MSCs.

Now that our attention was drawn to these 23 genes, we wanted to take a closer look at their behavior. First, we inspected their expression patterns in both species. We found that the 23 genes appeared to be upregulated in both the human and mouse MSC samples, except for PSMD4, which was downregulated in the mouse MSC samples compared to the TSC samples (Figures 4A, B). PSMD4 had a *p*-value of 6.27E-03 and a fold change of 0.136934557 in m_AT_MSCS, while in m_BM_MSCs, it had a *p*-value of 9.79E-03 and a fold change of –0.224675507. Since most genes were upregulated, we attempted to further explore the interplay between these genes and other systems that assist the proteasome in antioxidant defense, namely, antioxidant enzymes.

Consequently, members of the superoxide dismutase, glutathione peroxidase, peroxiredoxin, thioredoxin, and peroxidasin families were cross-referenced against the 1,583 common DEGs identified earlier. We found SOD3, GPX7, GPX8, PRDX2, PRDX4, TXN2, and PXDN were present in our list of common DEGs. The majority of the transcripts of these genes were upregulated but not all. Out of the seven genes, four (GPX7, GPX8, PXDN, TXN2) were upregulated in all analyzed MSC samples. Each of the other three (SOD3, PRDX2, and PRDX4) was upregulated in all the MSC samples except human AT-MSCs and BM-MSCs and mouse BM-MSCs, respectively (Figures 4C, D).

#### Gene interaction network

**FIGURE 5:**
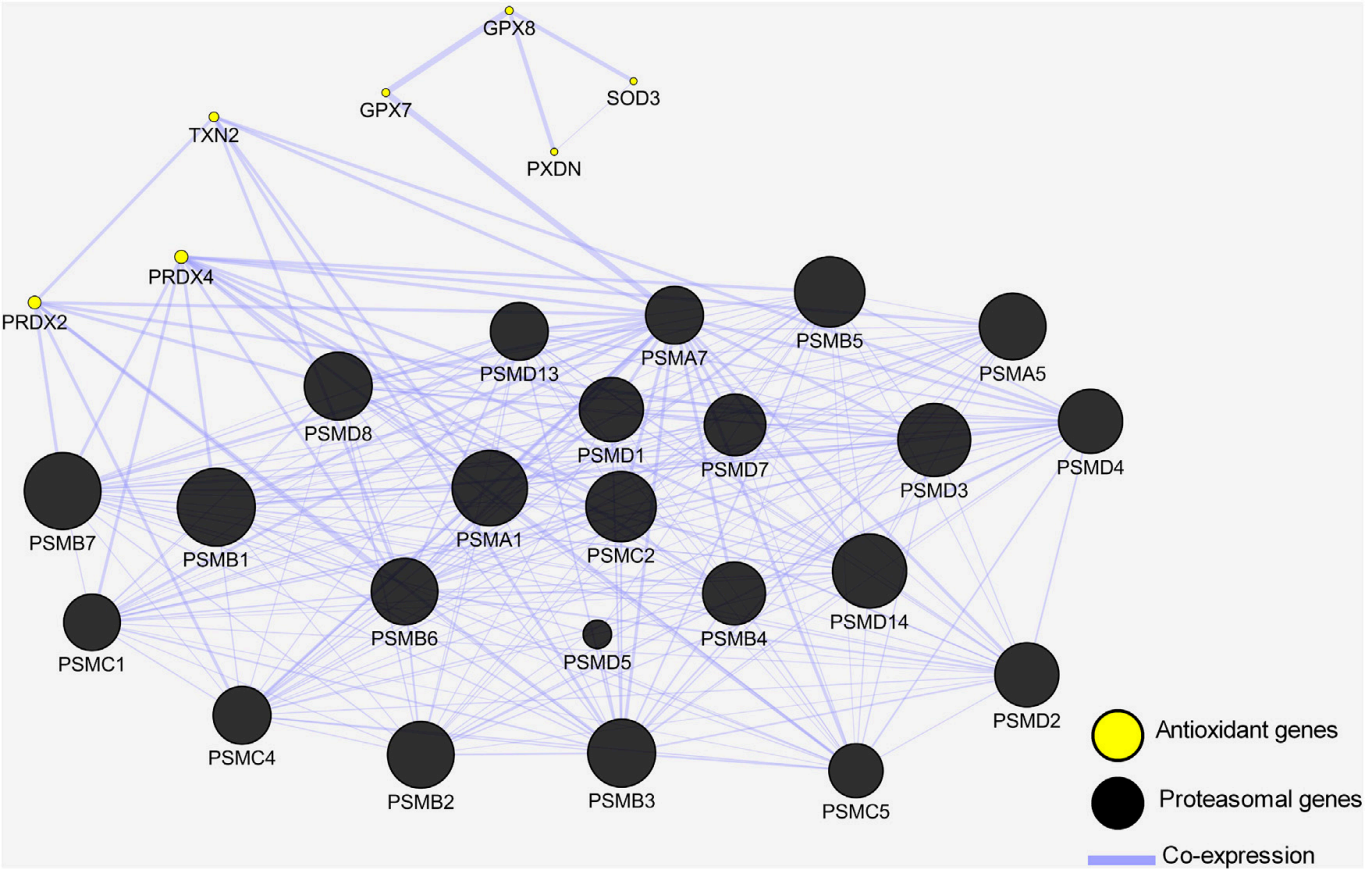
Gene interaction network shows antioxidant genes are co-expressed with proteasomal genes. Genes are depicted by nodes and the types of interaction are depicted by edges. Black circles represent proteasomal genes co-expressed with the antioxidant genes represented by yellow circles.

Next, we wanted to shed more light on the interaction between these antioxidant enzymes and the 23 members of the proteasomal degradation system identified earlier. We employed the help of Cytoscape and the Genemania database to understand the interplay between these antioxidant enzymes and the proteasomal genes. A gene interaction network was generated, and it showed all 23 genes of the proteasomal degradation pathway were co-expressed together and co-expressed with the antioxidant genes. Specifically, it showed PSMA7 to be co-expressed with GPX7, which in turn was co-expressed with GPX8, SOD3, and PXDN. Additionally, PRDX2 was co-expressed with PSMA7, PSMB3, PSMB6, PSMB7, PSMC4, PSMD3, and PSMD8. Furthermore, TXN2 was co-expressed with PSMA1, PSMA7, PSMB3, and PSMB6. Finally, PRDX4 was co-expressed with PSMA5, PSMB1, PSMB2, PSMB5, PSMB6, PSMC1, PSMC2, PSMC5, PSMD1, PSMD8, and PSMD14 (Figure 5).

#### Predictive model

Finally, we wanted to test the genes’ efficiency in predicting the identity of MSCs across the different tissue sources in both human and mouse species. To test this hypothesis, AutoWEKAClassifier performed 486 evaluations of available classifiers and found random forest to be the best classifier with the best error rate. The random forest tree classifier was used to train the model with 10-fold cross-validation, and the trained model was finalized. The final model was loaded to test its performance in predicting stem cell type on the testing data. We used the upregulated proteasomal genes as attributes, and we removed PSMD4 from the list since it had inconsistent expression across both species. We proceeded with the other 22 genes and ran the random forest model. The model tested the data 40 times and showed that MSCs were correctly classified in all 40 instances. To test whether all 22 genes contributed equally to the classification process, we ran the gain ratio attribute selection evaluator in WEKA. We found six genes to be the top contributors in the classification: PSMB5, PSMB1, PSMD14, PSMC4, PSMA1, and PSMD8 (Supplementary Table S7). We repeated the random forest model using these six genes and these six genes were enough to correctly classify the MSCs all 40 instances (Supplementary Table S8).

## Discussion

Ever since the discovery of MSCs by Friedenstein et al. (1968), researchers have debated their identity; however, the criteria proposed by the ISCT still fail to adequately describe MSCs and shows discrepancies across species and tissues of origin. As the focus shifted to stemness and stemness-related gene expression to aid in identifying MSCs, the search for adequate markers has intensified. Here, we show that members of the proteasome degradation system can be used as potential stemness-related markers to validate the identity of MSCs.

In this study, we integrated the RNA-seq data of MSCs derived from four different human tissues (AM, BM, AT, and UC) and two different mouse tissues (AT, BM). Differential expression analysis presented us with a list of 1,583 DEGs common to MSCs and TSCs across all tissue types in humans and mice. Further gene ontology enrichment analysis categorized these genes into GO terms, one of which was the GO term for regulating stem cell differentiation.

GO terms such as (GO:0055114) involved in oxidation-reduction process and GO term (GO:0006123) involved in mitochondrial electron transport, cytochrome c to oxygen were present in our results, however, they had higher frequencies than the GO term (GO:2000736) for stem cell regulation, which were 0.172%,0.044% and 0.010% respectively. Since a higher frequency denotes a more general term, we focused on the GOterm (GO:2000736) for stem cell regulation due to its uniqueness, high significance, and its specificity to stem cell processes. It included 23 members of the proteasome degradation system. Compelling evidence suggests a pivotal role for the proteasome in maintaining the pluripotency of mouse and human embryonic stem cells by supporting the clean-up of proteins oxidatively damaged during differentiation (Schröter and Adjaye, 2014).

The proteasome is an essential component of protein quality-control systems and plays a critical role in cellular homeostasis. It is involved in the degradation of abnormal, oxidized, or otherwise damaged proteins (Raynes et al., 2016). The accumulation of oxidized proteins in cells leads to their decreased life span (Reeg and Grune, 2015). Moreover, it has been demonstrated that dysfunction of the proteasome is heavily implicated in cell ageing (Chondrogianni et al., 2003). A recent study revealed that impairment of proteasome function resulted in an accumulation of oxidatively modified proteins in senescent Wharton’s jelly (WJ) and adipose-derived human adult mesenchymal stromal/stem cells. More importantly, this study showed that senescence of these cells’ is accompanied by a decline in proteasome content and activities, coupled with the concurrent loss of their stemness (Kapetanou et al., 2017). Although the degradation of oxidized proteins can occur by ubiquitin-dependent (26S-proteasome) and ubiquitin-independent (20S-proteasome) mechanisms (Shang et al., 2001; Goldberg, 2003; Bader et al., 2007), various studies have shown that the 20S proteasome might be the major machinery involved in this process (Silva et al., 2012; Jung et al., 2014). Here, we show that members of the 20S proteasome (PSMA1, PSMA5, and PSMA7) of the alpha subunits and all members of the beta subunit (PSMB1, PSMB2, PSMB3, PSMB4, PSMB5, PSMB6, and PSMB7) are not only differentially expressed in MSCs but are also upregulated.

Furthermore, we show that members of the 19S proteasome base (PSMC1, PSMC2, PSMC4, PSMC5, PSMD1, and PSMD2) and lid (PSMD3, PSMD5, PSMD7, PSMD8, PSMD13, and PSMD14) are also differentially expressed and upregulated in MSCs in comparison with TSCs. However, our results also showed that PSMD4 expression in mouse MSC samples was downregulated. PSMD4’s central role in the 19S lid is to recognize polyubiquitinated protein substrates and detach the ubiquitin molecules from them for their subsequent degradation through the 26S proteasomal pathway (da Fonseca et al., 2012). PSMD4 is not the only ubiquitin receptor in the 19S lid. PSMD2 is another ubiquitin receptor that recognizes and binds both ubiquitin and ubiquitin-like proteins (Chojnacki et al., 2017). We found PSMD2 to be upregulated in our mouse MSCs. It could be that mouse MSCs rely mainly on PSMD2 to recognize polyubiquitinated protein substrates, thereby rendering PSMD4 dispensable.

Studies have shown that during MSC proliferation, ROS are produced as byproducts of oxidative metabolism. However, increased ROS levels may lead to a decrease in cell survival and have also been implicated in cell senescence (Ko et al., 2012). To counteract these detrimental effects, the cell has antioxidant defense systems activated by high ROS levels. Increased ROS concentration causes Nrf2 (a stress-responsive transcription factor) to dissociate from its inhibitory complex with Keap1. This enables Nrf2 to accumulate and translocate to the nucleus, where it binds to antioxidant-response elements (ARE), thus promoting the expression of several antioxidant (Dai et al., 2020) and proteasomal genes (Kwak et al., 2003). These antioxidant enzymes and the proteasome degradation machinery work together as a defense against damaging high ROS levels. We demonstrated that members of the proteasome degradation machinery were upregulated across the MSCs samples tested. We also demonstrated that GPX7, GPX8, TXN2, and PXDN antioxidant genes were differentially expressed and upregulated in MSCs compared to TSCs. Additionally, we provided evidence that these genes are co-expressed with the proteasome degradation machinery members by data mining gene interaction databases. Taken together, these results point to the efficiency of MSCs in counteracting oxidative stress, in which the proteasome is integral.

Finally, to show the competence of these proteasome genes in validating the identity of MSCs, we employed the aid of predictive models. Predictive models have been used robustly to identify a general MSC phenotype that could distinguish MSCs from other cell types. A recent study showed that gene expression levels in prediction models increase the classification accuracy of the combined set of traditional MSC cell surface markers (Rohart et al., 2016). Using the random forest model, we showed that the expression of six proteasome genes could accurately distinguish MSCs from their tissue-specific counterparts. Of these six genes, PSMB5, PSMB1, and PSMD14 have been linked to stem cell function. As previously mentioned, PSMB1 and PSMB5 are catalytic subunits of the 20S proteasome, and reducing their expression leads to a decrease in cell proliferation and an increase in replicative senescence in hBMSCs (Yu et al., 2015). Likewise, Kapetanou and others reported a similar decline in the expression of these two genes in senescent WJ-MSCs. They also showed that PSMB5 overexpression rescues these senescent cells from age-related reductions in proteasome expression and function, improving their stemness and extending their lifespan (Kapetanou et al., 2017). Finally, PSMD14 is essential for proper 26S assembly (Quinet et al., 2020); it also plays a role in cleaving polyubiquitin chains at a proximal site and recycling ubiquitin chains (Lu et al., 2012). PSMD14 is a crucial regulator of stem cell maintenance. A reduction in its levels leads to a marked decrease in Oct4 protein expression, accompanied by abnormal morphology in embryonic stem cells (Buckley Shannon et al., 2012). However, no data currently exists on the three remaining genes (PSMC4, PSMA1, and PSMD8) that link them to any stem cell function.

Our study carried out a comprehensive comparative analysis of MSCs RNA-seq data across two species and six different tissue types to ascertain potential identity markers. Our results showed that six members of the proteasomal machinery are promising candidates for validating the identity of MSCs. Moreover, we shed a light on their association with antioxidant enzymes in defending MSCs against high ROS levels, thereby maintaining their proliferation and self-renewal. These six genes can be used as additional stemness-related markers to refine and enhance the accuracy of MSC identification, which is a critical step in ensuring the yield of a pure population for consequent applications in regenerative therapies.

Our prediction model is based only on MSCs and TSCs data for its training and testing sets; experimental validation of the expression levels of the six proposed stemness-related markers in MSCs from different sources, both on the RNA and protein levels, is crucial to confirm their efficiency in identifying MSCs. Experimental validation is beyond the scope of this study; however, it should be the focus of future confirmatory studies.

## Data Availability

The original contributions presented in the study are included in the article/Supplementary Material, further inquiries can be directed to the corresponding authors.
