# Supplementary material for: Computational comparative analysis identifies potential stemness-related markers for mesenchymal stromal/stem cells
Source: Front Cell Dev Biol. 2023 Mar 1;11:1065050. doi: 10.3389/fcell.2023.1065050 (PMC10014615; doi:10.3389/fcell.2023.1065050)
Supplement: Supplementary file 1 [file Table1.DOCX]

Supplementary table 1. Sample nomenclature

| **Sample name** | **Designated acronym/abbreviation** |
| --- | --- |
| Human umbilical cord derived mesenchymal stem cells | h_UC_MSCs |
| Human amnion derived mesenchymal stem cells | h_AM_MSCs |
| Human bone-marrow derived mesenchymal stem cells | h_BM_MSCs |
| Human adipose tissue derived mesenchymal stem cells | h_AT_MSCs |
| Human umbilical cord tissue specific cells | h_UC_TSCs |
| Human amnion tissue specific cells | h_AM_TSCs |
| Human bone-marrow tissue specific cells | h_BM_TSCs |
| Human adipose tissue specific cells | h_AT_TSCs |
| Mouse bone-marrow derived mesenchymal stem cells | m_BM_MSCs |
| Mouse adipose tissue derived mesenchymal stem cells | m_AT_MSCs |
| Mouse bone-marrow tissue specific cells | m_BM_TSCs |
| Mouse adipose tissue specific cells | m_AT_TSCs |
