# Supplementary material for: Computational comparative analysis identifies potential stemness-related markers for mesenchymal stromal/stem cells
Source: Front Cell Dev Biol. 2023 Mar 1;11:1065050. doi: 10.3389/fcell.2023.1065050 (PMC10014615; doi:10.3389/fcell.2023.1065050)
Supplement: Supplementary file 3 [file Table8.DOCX]

Supplementary table 8 Random Forest model results.

| **Instances** | **Predicted** | **Error prediction** |
| --- | --- | --- |
| 1 | MSC | 0.964 |
| 2 | MSC | 0.989 |
| 3 | MSC | 0.958 |
| 4 | MSC | 0.892 |
| 5 | MSC | 0.97 |
| 6 | MSC | 0.965 |
| 7 | MSC | 0.972 |
| 8 | MSC | 0.973 |
| 9 | MSC | 0.993 |
| 10 | MSC | 0.968 |
| 11 | MSC | 0.978 |
| 12 | MSC | 0.978 |
| 13 | MSC | 0.978 |
| 14 | MSC | 0.973 |
| 15 | MSC | 0.97 |
| 16 | MSC | 0.971 |
| 17 | MSC | 0.979 |
| 18 | MSC | 0.939 |
| 19 | MSC | 0.961 |
| 20 | MSC | 0.972 |
| 21 | TSC | 0.975 |
| 22 | TSC | 0.97 |
| 23 | TSC | 0.971 |
| 24 | TSC | 0.96 |
| 25 | TSC | 0.97 |
| 26 | TSC | 0.981 |
| 27 | TSC | 0.948 |
| 28 | TSC | 0.907 |
| 29 | TSC | 0.988 |
| 30 | TSC | 0.993 |
| 31 | TSC | 0.98 |
| 32 | TSC | 0.977 |
| 33 | TSC | 0.961 |
| 34 | TSC | 0.97 |
| 35 | TSC | 0.972 |
| 36 | TSC | 0.908 |
| 37 | TSC | 0.98 |
| 38 | TSC | 0.97 |
| 39 | TSC | 0.976 |
| 40 | TSC | 0.97 |
