# Supplementary figures and images for: Computational comparative analysis identifies potential stemness-related markers for mesenchymal stromal/stem cells
Source: Front Cell Dev Biol. 2023 Mar 1;11:1065050. doi: 10.3389/fcell.2023.1065050 (PMC10014615; doi:10.3389/fcell.2023.1065050)

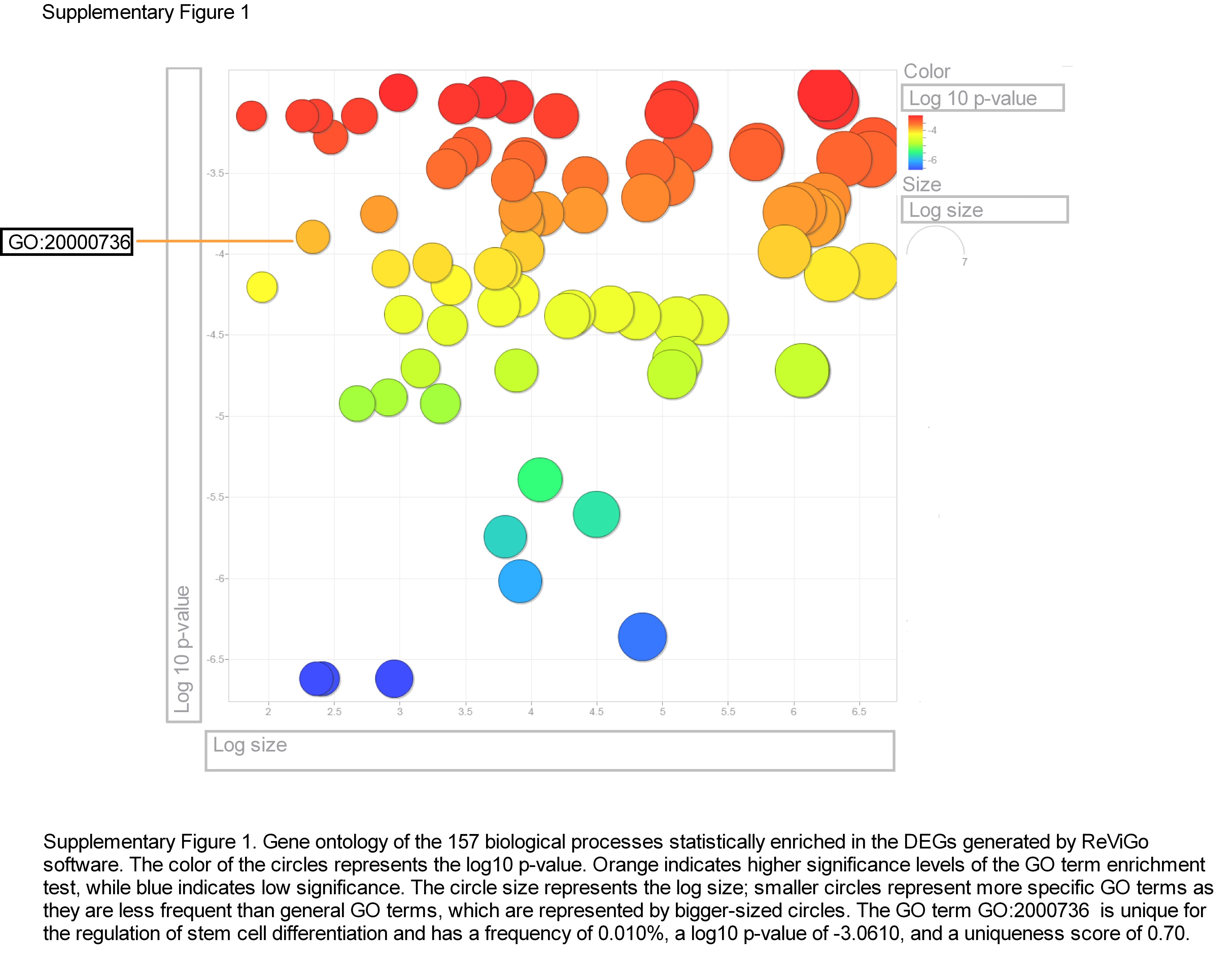

Supplement: Supplementary file 5 [file Image1.JPEG]
