# Supplementary material for: Computational comparative analysis identifies potential stemness-related markers for mesenchymal stromal/stem cells
Source: Front Cell Dev Biol. 2023 Mar 1;11:1065050. doi: 10.3389/fcell.2023.1065050 (PMC10014615; doi:10.3389/fcell.2023.1065050)
Supplement: Supplementary file 6 [file Table7.DOCX]

Supplementary table 7 Gain Ratio Attribute selection results.

| **Score** | **Attribute name** |
| --- | --- |
| 1 | PSMB5 |
| 1 | PSMC4 |
| 1 | PSMA1 |
| 1 | PSMD8 |
| 1 | PSMB1 |
| 1 | PSMD14 |
| 0.8565427071334994 | PSMB3 |
| 0.8565427071334994 | PSMC2 |
| 0.7637954966984674 | PSMD5 |
| 0.6900298961146358 | PSMD3 |
| 0.6900298961146358 | PSMD7 |
| 0.628236442198776 | PSMC1 |
| 0.6216210833140299 | PSMB6 |
| 0.5749951688786842 | PSMB2 |
| 0.5749951688786842 | PSMB4 |
| 0.5749951688786842 | PSMC5 |
| 0.5486199014124902 | PSMD1 |
| 0.5282512370705057 | PSMA7 |
| 0.5282512370705057 | PSMA5 |
| 0.5282512370705057 | PSMD2 |
| 0.47804641202037645 | PSMD13 |
| 0.4426762971573124 | PSMB7 |
