# Supplementary material for: Computational comparative analysis identifies potential stemness-related markers for mesenchymal stromal/stem cells
Source: Front Cell Dev Biol. 2023 Mar 1;11:1065050. doi: 10.3389/fcell.2023.1065050 (PMC10014615; doi:10.3389/fcell.2023.1065050)
Supplement: Supplementary file 7 [file Table2.docx]

Supplementary table 2 Datasets.

| Species | Tissue type | File | Database/Acession | References |
| --- | --- | --- | --- | --- |
| Human | AM-MSCs | SRR7724016  SRR7724017  SRR7724018  SRR7724019 | GEO  GSE118808 | (1) |
|  | UC-MSCs | SRR7724024  SRR7724025  SRR7724026  SRR7724027 | GEO  GSE118808 | (1) |
|  | AT-MSCS | SRR5357813  SRR5357815  SRR5357817 | GEO  GSE96788 | (2) |
|  | BM-MSCs | SRR6189571  SRR6189572  SRR6189573 | GEO GSE105145 | (3) |
|  | AM-TSCs | SRR3317121  SRR3317142  SRR3317145  SRR3317146 | GEO  GSE79782 | (4) |
|  | UC-TSCs | SRR6217829  SRR6217830  SRR6217831 | GEO  GSE106170 | (5) |
|  | AT-TSCs | ERR315332  ERR315343  ERR315378 | Array express  E-MTAB-1733 | (6) |
|  | BM-TSCs | ERR315395  ERR315396  ERR315406 | Array express  E-MTAB-1733 | (6) |
| Mouse | AT-MSCS | SRR7465589  SRR7465590  SRR7465591 | GEO  GSE116558 | (7) |
|  | BM-MSCs | SRR7465598  SRR7465599  SRR7465600 | GEO  GSE116558 | (7) |
|  | AT-TSCs | SRR10071341  SRR10071342  SRR10071343 | GEO  GSE136912 | (8) |
|  | BM-TSCs | SRR5273648  SRR5273664  SRR5273680 | GEO  GSE118808 | (9) |

Referenences

1. Ma J, Wu J, Han L, Jiang X, Yan L, Hao J, et al. Comparative analysis of mesenchymal stem cells derived from amniotic membrane, umbilical cord, and chorionic plate under serum-free condition. Stem Cell Res Ther. 2019;10(1):19.

2. Samsonraj RM, Dudakovic A, Manzar B, Sen B, Dietz AB, Cool SM, et al. Osteogenic Stimulation of Human Adipose-Derived Mesenchymal Stem Cells Using a Fungal Metabolite That Suppresses the Polycomb Group Protein EZH2. Stem Cells Transl Med. 2018;7(2):197-209.

3. Shen W-C, Lai Y-C, Li L-H, Liao K, Lai H-C, Kao S-Y, et al. Methylation and PTEN activation in dental pulp mesenchymal stem cells promotes osteogenesis and reduces oncogenesis. Nat Commun [Internet]. 2019 2019/05//; 10(1):[2226 p.]. Available from: <http://europepmc.org/abstract/MED/31110221>

<https://doi.org/10.1038/s41467-019-10197-x>

<https://europepmc.org/articles/PMC6527698>

<https://europepmc.org/articles/PMC6527698?pdf=render>.

4. Suzuki M, Maekawa R, Patterson NE, Reynolds DM, Calder BR, Reznik SE, et al. Amnion as a surrogate tissue reporter of the effects of maternal preeclampsia on the fetus. Clinical Epigenetics. 2016;8(1):67.

5. Heshmati Y, Kharazi S, Türköz G, Chang D, Kamali Dolatabadi E, Boström J, et al. The histone chaperone NAP1L3 is required for haematopoietic stem cell maintenance and differentiation. Scientific Reports. 2018;8(1):11202.

6. Fagerberg L, Hallström BM, Oksvold P, Kampf C, Djureinovic D, Odeberg J, et al. Analysis of the human tissue-specific expression by genome-wide integration of transcriptomics and antibody-based proteomics. Molecular & cellular proteomics : MCP. 2014;13(2):397-406.

7. Ho Y-T, Shimbo T, Wijaya E, Ouchi Y, Takaki E, Yamamoto R, et al. Chromatin accessibility identifies diversity in mesenchymal stem cells from different tissue origins. Scientific Reports. 2018;8(1):17765.

8. Tang X, Miao Y, Luo Y, Sriram K, Qi Z, Lin FM, et al. Suppression of Endothelial AGO1 Promotes Adipose Tissue Browning and Improves Metabolic Dysfunction. Circulation. 2020;142(4):365-79.

9. Shen Y, Yue F, McCleary DF, Ye Z, Edsall L, Kuan S, et al. A map of the cis-regulatory sequences in the mouse genome. Nature. 2012;488(7409):116-20.
