# Supplementary material for: Computational comparative analysis identifies potential stemness-related markers for mesenchymal stromal/stem cells
Source: Front Cell Dev Biol. 2023 Mar 1;11:1065050. doi: 10.3389/fcell.2023.1065050 (PMC10014615; doi:10.3389/fcell.2023.1065050)
Supplement: Supplementary file 8 [file Table6.DOCX]

Supplementary table 6 GOrilla and ReViGO generated GO terms.

| GO term | Process | Frequency | Uniqueness | P value | Q value |
| --- | --- | --- | --- | --- | --- |
| GO:0009987 | cellular process | 78.03769 | 1 | 1.03E-4 | 8.86E-3 |
| GO:0007160 | cell-matrix adhesion | 0.040129 | 0.995369 | 1.03E-4 | 8.86E-3 |
| GO:0071840 | cellular component organization or biogenesis | 9.051042 | 0.992057 | 1.93E-4 | 1.35E-2 |
| GO:1901998 | toxin transport | 0.014496 | 0.990192 | 1.39E-4 | 1.14E-2 |
| GO:0044281 | small molecule metabolic process | 15.77423 | 0.988976 | 5.24E-5 | 6.1E-3 |
| GO:0003013 | circulatory system process | 0.092048 | 0.988512 | 4.69E-4 | 2.59E-2 |
| GO:0043588 | skin development | 0.050743 | 0.987927 | 4.71E-5 | 5.84E-3 |
| GO:0035987 | endodermal cell differentiation | 0.007598 | 0.984535 | 1.9E-5 | 3.48E-3 |
| GO:0006891 | intra-Golgi vesicle-mediated transport | 0.039917 | 0.977977 | 7E-7 | 4.89E-4 |
| GO:0019646 | aerobic electron transport chain | 0.173494 | 0.976611 | 8.18E-5 | 7.66E-3 |
| GO:0031146 | SCF-dependent proteasomal ubiquitin-dependent protein catabolic process | 0.044474 | 0.976609 | 8.1E-4 | 3.55E-2 |
| GO:0006888 | endoplasmic reticulum to Golgi vesicle-mediated transport | 0.161678 | 0.976502 | 3.12E-4 | 1.92E-2 |
| GO:1902600 | proton transmembrane transport | 1.099905 | 0.974922 | 1.93E-4 | 1.35E-2 |
| GO:0007339 | binding of sperm to zona pellucida | 0.010598 | 0.974189 | 4.38E-6 | 1.68E-3 |
| GO:0098662 | inorganic cation transmembrane transport | 2.282271 | 0.973881 | 1.3E-5 | 2.78E-3 |
| GO:0044770 | cell cycle phase transition | 0.050125 | 0.971086 | 3.03E-4 | 1.91E-2 |
| GO:0043062 | extracellular structure organization | 0.065475 | 0.970883 | 1.57E-4 | 1.22E-2 |
| GO:0018212 | peptidyl-tyrosine modification | 0.223081 | 0.969844 | 4.36E-4 | 3.5E-2 |
| GO:0044772 | mitotic cell cycle phase transition | 0.048137 | 0.969654 | 2.12E-4 | 1.43E-2 |
| GO:0022402 | cell cycle process | 0.845822 | 0.96894 | 1.83E-4 | 1.31E-2 |
| GO:0044249 | cellular biosynthetic process | 22.78187 | 0.967897 | 4.66E-4 | 2.6E-2 |
| GO:0016310 | phosphorylation | 7.702117 | 0.966527 | 3.59E-4 | 2.12E-2 |
| GO:0030199 | collagen fibril organization | 0.011691 | 0.966446 | 9.15E-4 | 3.84E-2 |
| GO:1903047 | mitotic cell cycle process | 0.327126 | 0.966341 | 3.61E-5 | 5.05E-3 |
| GO:0006631 | fatty acid metabolic process | 1.130463 | 0.964556 | 7.16E-4 | 3.39E-2 |
| GO:0006796 | phosphate-containing compound metabolic process | 13.8766 | 0.964281 | 1.89E-4 | 1.33E-2 |
| GO:0030198 | extracellular matrix organization | 0.06376 | 0.963586 | 5.53E-5 | 6.25E-3 |
| GO:0016043 | cellular component organization | 7.326581 | 0.962258 | 9.77E-5 | 8.53E-3 |
| GO:0018108 | peptidyl-tyrosine phosphorylation | 0.209455 | 0.962005 | 4.36E-4 | 3.53E-2 |
| GO:0006082 | organic acid metabolic process | 9.213416 | 0.960113 | 2.44E-5 | 3.91E-3 |
| GO:0009612 | response to mechanical stimulus | 0.029034 | 0.9597 | 4.71E-5 | 5.84E-3 |
| GO:0006123 | mitochondrial electron transport, cytochrome c to oxygen | 0.000433 | 0.980 | 8.18E-5 | 7.75E-3 |
| GO:0019882 | antigen processing and presentation | 0.033583 | 0.957031 | 5.69E-4 | 2.92E-2 |
| GO:0051385 | response to mineralocorticoid | 0.001471 | 0.955279 | 7.91E-4 | 3.55E-2 |
| GO:0019752 | carboxylic acid metabolic process | 8.848734 | 0.95499 | 7.67E-5 | 7.55E-3 |
| GO:0055114 | oxidation-reduction process | 0.000172 | 0.964 | 9.14E-4 | 3.86E-2 |
| GO:0005513 | detection of calcium ion | 0.00133 | 0.953333 | 8.14E-5 | 5.67E-3 |
| GO:0050982 | detection of mechanical stimulus | 0.009657 | 0.951596 | 1.77E-4 | 1.31E-2 |
| GO:0001975 | response to amphetamine | 0.003236 | 0.950449 | 8.14E-5 | 5.73E-3 |
| GO:0001732 | formation of cytoplasmic translation initiation complex | 0.074652 | 0.949195 | 8.42E-4 | 2.7E-2 |
| GO:0006415 | translational termination | 0.176518 | 0.943213 | 2.76E-4 | 1.78E-2 |
| GO:0043200 | response to amino acid | 0.022235 | 0.94299 | 8.12E-4 | 3.54E-2 |
| GO:0019886 | antigen processing and presentation of exogenous peptide antigen via MHC class II | 0.002527 | 0.94122 | 1.3E-5 | 2.78E-3 |
| GO:0002495 | antigen processing and presentation of peptide antigen via MHC class II | 0.002896 | 0.941148 | 1.3E-5 | 2.86E-3 |
| GO:0002504 | antigen processing and presentation of peptide or polysaccharide antigen via MHC class II | 0.008783 | 0.94106 | 1.3E-5 | 2.94E-3 |
| GO:0019884 | antigen processing and presentation of exogenous antigen | 0.011132 | 0.940456 | 8.84E-4 | 3.75E-2 |
| GO:0048002 | antigen processing and presentation of peptide antigen | 0.013763 | 0.939909 | 4.45E-4 | 2.53E-2 |
| GO:0002478 | antigen processing and presentation of exogenous peptide antigen | 0.006463 | 0.939427 | 8.84E-4 | 3.77E-2 |
| GO:0009593 | detection of chemical stimulus | 0.363373 | 0.936989 | 8.14E-5 | 5.52E-3 |
| GO:0071230 | cellular response to amino acid stimulus | 0.019111 | 0.936471 | 2.91E-4 | 1.85E-2 |
| GO:1901844 | regulation of cell communication by electrical coupling involved in cardiac conduction | 0.000845 | 0.826683 | 5.99E-5 | 6.48E-3 |
| GO:0051343 | positive regulation of cyclic-nucleotide phosphodiesterase activity | 0.000427 | 0.824581 | 8.14E-5 | 5.57E-3 |
| GO:0010649 | regulation of cell communication by electrical coupling | 0.001123 | 0.824155 | 3.05E-4 | 1.91E-2 |
| GO:0007190 | activation of adenylate cyclase activity | 0.003244 | 0.807013 | 2E-4 | 1.36E-2 |
| GO:1902036 | regulation of hematopoietic stem cell differentiation | 0.005771 | 0.790935 | 1.21E-4 | 1.01E-2 |
| GO:0090175 | regulation of establishment of planar polarity | 0.009214 | 0.789441 | 3.23E-4 | 1.97E-2 |
| GO:1903522 | regulation of blood circulation | 0.048912 | 0.788886 | 5.59E-5 | 6.22E-3 |
| GO:1904872 | regulation of telomerase RNA localization to Cajal body | 0.0014 | 0.77592 | 1.18E-5 | 2.82E-3 |
| GO:0032886 | regulation of microtubule-based process | 0.090569 | 0.775322 | 1.74E-5 | 3.35E-3 |
| GO:0033238 | regulation of cellular amine metabolic process | 0.01856 | 0.773656 | 5.02E-5 | 5.93E-3 |
| GO:0032101 | regulation of response to external stimulus | 0.228189 | 0.771213 | 3.73E-5 | 5.03E-3 |
| GO:0035567 | non-canonical Wnt signaling pathway | 0.008597 | 0.770507 | 8.24E-5 | 7.63E-3 |
| GO:0098901 | regulation of cardiac muscle cell action potential | 0.004048 | 0.769829 | 1.48E-5 | 3.06E-3 |
| GO:0006521 | regulation of cellular amino acid metabolic process | 0.016203 | 0.76867 | 5.02E-5 | 6.02E-3 |
| GO:1904869 | regulation of protein localization to Cajal body | 0.000423 | 0.765985 | 2.35E-7 | 9.02E-4 |
| GO:1903706 | regulation of hemopoiesis | 0.058498 | 0.763704 | 2.3E-4 | 1.54E-2 |
| GO:0098900 | regulation of action potential | 0.007047 | 0.762668 | 7E-7 | 4.89E-4 |
| GO:0062012 | regulation of small molecule metabolic process | 0.107397 | 0.75653 | 9.33E-6 | 2.47E-3 |
| GO:0042127 | regulation of cell population proliferation | 0.299406 | 0.755002 | 7.89E-5 | 7.67E-3 |
| GO:0051239 | regulation of multicellular organismal process | 0.552606 | 0.754955 | 1.47E-4 | 1.16E-2 |
| GO:0010565 | regulation of cellular ketone metabolic process | 0.050585 | 0.752621 | 2.57E-6 | 1.16E-3 |
| GO:0006446 | regulation of translational initiation | 0.046364 | 0.750673 | 9.44E-4 | 3.92E-2 |
| GO:0060071 | Wnt signaling pathway, planar cell polarity pathway | 0.006355 | 0.749864 | 3.23E-4 | 1.95E-2 |
| GO:0070202 | regulation of establishment of protein localization to chromosome | 0.002237 | 0.749753 | 2.35E-7 | 3.01E-4 |
| GO:0050821 | protein stabilization | 0.043708 | 0.749465 | 1.05E-4 | 8.97E-3 |
| GO:1904814 | regulation of protein localization to chromosome, telomeric region | 0.002606 | 0.748841 | 1.25E-6 | 6.84E-4 |
| GO:2000104 | negative regulation of DNA-dependent DNA replication | 0.018312 | 0.745392 | 3.09E-4 | 1.91E-2 |
| GO:0070203 | regulation of establishment of protein localization to telomere | 0.002026 | 0.743162 | 2.35E-7 | 3.61E-4 |
| GO:0031647 | regulation of protein stability | 0.064091 | 0.743146 | 4.63E-4 | 2.62E-2 |
| GO:0051726 | regulation of cell cycle | 0.551342 | 0.743072 | 4E-7 | 3.42E-4 |
| GO:0008156 | negative regulation of DNA replication | 0.024688 | 0.740856 | 8.33E-4 | 3.59E-2 |
| GO:1904874 | positive regulation of telomerase RNA localization to Cajal body | 0.000791 | 0.739192 | 1.18E-5 | 2.92E-3 |
| GO:0007229 | integrin-mediated signaling pathway | 0.085672 | 0.738488 | 4.85E-4 | 2.65E-2 |
| GO:0050793 | regulation of developmental process | 1.301765 | 0.736709 | 2.69E-4 | 1.78E-2 |
| GO:0051052 | regulation of DNA metabolic process | 0.157266 | 0.736364 | 8.34E-6 | 2.46E-3 |
| GO:0051128 | regulation of cellular component organization | 0.915457 | 0.732199 | 5.2E-6 | 1.66E-3 |
| GO:0045595 | regulation of cell differentiation | 0.287847 | 0.731719 | 5.95E-4 | 3.03E-2 |
| GO:1901841 | regulation of high voltage-gated calcium channel activity | 0.003372 | 0.726989 | 1.68E-4 | 1.27E-2 |
| GO:1904871 | positive regulation of protein localization to Cajal body | 0.000423 | 0.723879 | 2.35E-7 | 4.51E-4 |
| GO:0046889 | positive regulation of lipid biosynthetic process | 0.014144 | 0.723692 | 7.7E-4 | 3.52E-2 |
| GO:2000134 | negative regulation of G1/S transition of mitotic cell cycle | 0.014587 | 0.722107 | 9.77E-4 | 3.99E-2 |
| GO:1902807 | negative regulation of cell cycle G1/S phase transition | 0.015921 | 0.721573 | 9.77E-4 | 4.01E-2 |
| GO:2000278 | regulation of DNA biosynthetic process | 0.025591 | 0.721473 | 4.5E-5 | 5.76E-3 |
| GO:0022603 | regulation of anatomical structure morphogenesis | 0.898226 | 0.719355 | 1.67E-4 | 1.27E-2 |
| GO:1900180 | regulation of protein localization to nucleus | 0.021755 | 0.71908 | 4.18E-4 | 2.4E-2 |
| GO:0051174 | regulation of phosphorus metabolic process | 0.447984 | 0.718697 | 9.08E-5 | 8.11E-3 |
| GO:1901385 | regulation of voltage-gated calcium channel activity | 0.007267 | 0.717787 | 4.98E-4 | 2.66E-2 |
| GO:0010923 | negative regulation of phosphatase activity | 0.016041 | 0.713874 | 3.34E-4 | 1.99E-2 |
| GO:1901842 | negative regulation of high voltage-gated calcium channel activity | 0.002722 | 0.713173 | 8.14E-5 | 5.62E-3 |
| GO:0045785 | positive regulation of cell adhesion | 0.067812 | 0.712172 | 7.53E-5 | 7.51E-3 |
| GO:0001558 | regulation of cell growth | 0.078633 | 0.705456 | 2.06E-5 | 3.59E-3 |
| GO:1901386 | negative regulation of voltage-gated calcium channel activity | 0.004897 | 0.704729 | 2.42E-5 | 3.96E-3 |
| GO:0007346 | regulation of mitotic cell cycle | 0.18705 | 0.70322 | 1.79E-4 | 1.31E-2 |
| GO:0007204 | positive regulation of cytosolic calcium ion concentration | 0.048783 | 0.701677 | 5.56E-7 | 4.27E-4 |
| GO:2000736 | regulation of stem  differentiation | 0.010213 | 0.70021 | 8.69E-4 | 4.64E-2 |
| GO:0001932 | regulation of protein phosphorylation | 0.288245 | 0.699967 | 1.82E-4 | 1.32E-2 |
| GO:0051480 | regulation of cytosolic calcium ion concentration | 0.055213 | 0.699328 | 1.78E-7 | 1.37E-3 |
| GO:1904851 | positive regulation of establishment of protein localization to telomere | 0.001864 | 0.69919 | 2.35E-7 | 6.02E-4 |
| GO:2001258 | negative regulation of cation channel activity | 0.008858 | 0.69878 | 1.49E-4 | 1.17E-2 |
| GO:0051783 | regulation of nuclear division | 0.067745 | 0.697245 | 4.06E-5 | 5.28E-3 |
| GO:1904816 | positive regulation of protein localization to chromosome, telomeric region | 0.002233 | 0.697188 | 1.25E-6 | 7.37E-4 |
| GO:0019220 | regulation of phosphate metabolic process | 0.447744 | 0.694845 | 9.08E-5 | 8.2E-3 |
| GO:0007166 | cell surface receptor signaling pathway | 0.944868 | 0.694502 | 2.19E-5 | 3.74E-3 |
| GO:0070507 | regulation of microtubule cytoskeleton organization | 0.065823 | 0.693284 | 1.74E-5 | 3.27E-3 |
| GO:0072507 | divalent inorganic cation homeostasis | 0.144506 | 0.692585 | 1.55E-6 | 7.95E-4 |
| GO:0010564 | regulation of cell cycle process | 0.354457 | 0.689596 | 5.17E-6 | 1.73E-3 |
| GO:0055074 | calcium ion homeostasis | 0.116508 | 0.689469 | 3E-7 | 2.88E-4 |
| GO:0007186 | G protein-coupled receptor signaling pathway | 1.166478 | 0.689174 | 9.66E-4 | 3.99E-2 |
| GO:0033044 | regulation of chromosome organization | 0.112957 | 0.687199 | 5.68E-4 | 2.93E-2 |
| GO:0006874 | cellular calcium ion homeostasis | 0.111499 | 0.685328 | 3E-7 | 3.29E-4 |
| GO:0072503 | cellular divalent inorganic cation homeostasis | 0.119101 | 0.684568 | 1.55E-6 | 7.46E-4 |
| GO:0010948 | negative regulation of cell cycle process | 0.150165 | 0.682922 | 8.81E-4 | 2.8E-2 |
| GO:2000573 | positive regulation of DNA biosynthetic process | 0.016406 | 0.675788 | 1.95E-4 | 1.34E-2 |
| GO:0060236 | regulation of mitotic spindle organization | 0.017417 | 0.673085 | 7.61E-4 | 3.52E-2 |
| GO:0090224 | regulation of spindle organization | 0.018316 | 0.672154 | 7.61E-4 | 3.55E-2 |
| GO:1900182 | positive regulation of protein localization to nucleus | 0.014123 | 0.671774 | 7.28E-4 | 3.41E-2 |
| GO:0032204 | regulation of telomere maintenance | 0.019998 | 0.67107 | 5.18E-4 | 2.72E-2 |
| GO:1904356 | regulation of telomere maintenance via telomere lengthening | 0.013303 | 0.670651 | 2.85E-5 | 4.38E-3 |
| GO:0006875 | cellular metal ion homeostasis | 0.304813 | 0.668922 | 9.85E-6 | 2.52E-3 |
| GO:0032210 | regulation of telomere maintenance via telomerase | 0.012226 | 0.668694 | 4.65E-6 | 1.62E-3 |
| GO:0055065 | metal ion homeostasis | 0.41465 | 0.668684 | 1.55E-5 | 3.13E-3 |
| GO:0051493 | regulation of cytoskeleton organization | 0.269076 | 0.668527 | 6.02E-4 | 3.04E-2 |
| GO:0019222 | regulation of metabolic process | 13.46548 | 0.668226 | 2.74E-4 | 1.78E-2 |
| GO:0030003 | cellular cation homeostasis | 0.327777 | 0.668203 | 2.38E-5 | 3.97E-3 |
| GO:0050801 | ion homeostasis | 0.537778 | 0.667642 | 7.91E-5 | 7.59E-3 |
| GO:0006873 | cellular ion homeostasis | 0.369032 | 0.666806 | 2.95E-5 | 4.37E-3 |
| GO:0050789 | regulation of biological process | 21.76506 | 0.666755 | 6.06E-4 | 3.04E-2 |
| GO:0055080 | cation homeostasis | 0.483245 | 0.66639 | 3.71E-5 | 5.09E-3 |
| GO:0033043 | regulation of organelle organization | 0.498263 | 0.665922 | 2.87E-5 | 4.32E-3 |
| GO:0098771 | inorganic ion homeostasis | 0.521575 | 0.664479 | 4.62E-5 | 5.82E-3 |
| GO:0051054 | positive regulation of DNA metabolic process | 0.054616 | 0.66016 | 6.57E-4 | 3.28E-2 |
| GO:2001252 | positive regulation of chromosome organization | 0.022616 | 0.654741 | 5.84E-5 | 6.4E-3 |
| GO:0000079 | regulation of cyclin-dependent protein serine/threonine kinase activity | 0.077726 | 0.653963 | 3.9E-6 | 1.66E-3 |
| GO:1904029 | regulation of cyclin-dependent protein kinase activity | 0.078451 | 0.653762 | 8.81E-6 | 2.51E-3 |
| GO:0007088 | regulation of mitotic nuclear division | 0.057135 | 0.653145 | 8.54E-5 | 7.81E-3 |
| GO:0050794 | regulation of cellular process | 19.87407 | 0.65304 | 2.7E-4 | 1.77E-2 |
| GO:0045787 | positive regulation of cell cycle | 0.087735 | 0.652037 | 3E-5 | 4.35E-3 |
| GO:1904358 | positive regulation of telomere maintenance via telomere lengthening | 0.008406 | 0.637352 | 1.18E-5 | 2.74E-3 |
| GO:0032212 | positive regulation of telomere maintenance via telomerase | 0.007988 | 0.636582 | 4.65E-6 | 1.7E-3 |
| GO:0048522 | positive regulation of cellular process | 1.92505 | 0.634109 | 1.84E-4 | 1.31E-2 |
| GO:0007165 | signal transduction | 7.216175 | 0.633819 | 3.57E-5 | 5.07E-3 |
| GO:0080090 | regulation of primary metabolic process | 12.36607 | 0.632658 | 1.76E-4 | 1.31E-2 |
| GO:0009893 | positive regulation of metabolic process | 1.407974 | 0.631539 | 7.19E-6 | 2.21E-3 |
| GO:0032206 | positive regulation of telomere maintenance | 0.013547 | 0.628835 | 1.95E-4 | 1.35E-2 |
| GO:0051173 | positive regulation of nitrogen compound metabolic process | 1.254689 | 0.627316 | 3.78E-5 | 5E-3 |
| GO:0010604 | positive regulation of macromolecule metabolic process | 1.329005 | 0.627056 | 1.65E-5 | 3.24E-3 |
| GO:0031325 | positive regulation of cellular metabolic process | 1.311319 | 0.617988 | 9.29E-6 | 2.55E-3 |
